# Antibiotic and antiviral prescribing patterns among hospitalised patients with COVID-19 pneumonia in Mongolia: a real-world descriptive retrospective study

**DOI:** 10.1080/20523211.2026.2731647

**Published:** 2026-09-18

**Authors:** Narangarav Nyamdag, Nina Muratkhan, Munkhbat Sukhee, Erdenetuya Myagmarsuren

**Affiliations:** a School of Pharmacy, Mongolian National University of Medical Sciences, Ulaanbaatar, Mongolia; b Mongolia-Japan Hospital, Mongolian National University of Medical Sciences, Ulaanbaatar, Mongolia

**Keywords:** COVID-19 pneumonia, antibiotic prescribing, antibiotic stewardship, low- and middle-income countries (LMICs), health system resilience

## Abstract

**Background:**

During the COVID-19 pandemic, Mongolia, like many low- and middle-income countries, faced major healthcare delivery challenges due to limited system readiness, resource constraints, geopolitical vulnerabilities, and prolonged border closures. These factors caused shortages of laboratory reagents and globally recommended therapeutics, including remdesivir and tocilizumab, prompting clinicians to use alternative antivirals and empirical broad-spectrum antibiotics.

**Objective:**

To evaluate and compare real-world prescribing patterns of antibiotics and antivirals among hospitalised patients with COVID-19 pneumonia at Mongolia’s two largest tertiary referral facilities: Mongolia-Japan Hospital (MJH) and the National Center for Communicable Diseases (NCCD).

**Methods:**

This retrospective cross-sectional study analysed anonymised medical charts and electronic health records of 1012 adults with PCR-confirmed COVID-19 pneumonia hospitalised between 18 April and 31 December 2021. Demographics, comorbidities, laboratory markers, and pharmacological treatments were systematically extracted and compared between the two institutions.

**Results:**

Antibiotics were prescribed to 87.6% (887/1012) of patients. Cephalosporins were the most frequently prescribed class (89.3%, 904/1012), followed by macrolides (36.5%, 369/1012) and fluoroquinolones (33.5%, 339/1012). Multi-drug empiricism was common, with beta-lactam, macrolide, and quinolone combinations accounting for 70.8% (68/96) of triple-antibiotic regimens. Antivirals were administered to over 90% of patients, predominantly umifenovir (52.2%) and favipiravir, supported by domestic stockpiles and Eurasian trade networks. Remdesivir was rarely used, while tocilizumab was administered to only 2 patients (0.2%). Hypertension and diabetes were significantly more frequent in the MJH cohort, reflecting differences in catchment populations and screening practices.

**Conclusion:**

The high rate of empirical broad-spectrum antibiotic use and reliance on alternative antivirals reflect pragmatic clinical adaptations to diagnostic reagent shortages and therapeutic supply inequities. These findings highlight the need for national antimicrobial stewardship programmes, strengthened microbiological diagnostic capacity, and digitised clinical registries to improve healthcare system resilience in resource-constrained settings.

## Background

The COVID-19 pandemic had a profoundly disruptive effect on global health systems, exposing systemic weaknesses and challenging the resilience of health services in both high-income and low-income countries (Haldane et al., 2021; Ibn-Mohammed et al., 2021; World Health Organization [WHO], 2020a). In Mongolia, early efforts to contain the virus were undermined by considerable difficulties in maintaining essential health services and delivering coordinated patient care across successive waves of infection (Bayasgalan et al., 2021; Dambadarjaa et al., 2022). Although the country initially recorded relatively few cases, the absence of a dedicated public health emergency system, such as a nationally mandated health protection agency, limited its capacity to respond rapidly and effectively (Dambadarjaa et al., 2022; Guan et al., 2020; Huang et al., 2021; Ministry of Health [MOH], 2023). The response was further weakened by fragmented health information systems, insufficient financing, and a shortage of personal protective equipment for front-line health workers (Chen et al., 2020; MOH, 2023).

The pandemic also acted as a catalyst for rapid gains in health infrastructure, vaccination coverage, and inter-sectoral coordination. By early 2022, primary-series vaccination coverage in Mongolia exceeded 70%, and more than 30% of the population had received a booster dose (Dambadarjaa et al., 2022; MOH, 2023; WHO, 2022a).

Despite these gains, the prolonged course of the pandemic has continued to pose challenges, including the emergence of new viral strains, the ongoing burden of post-infection complications, and sustained disruption of essential health services (Arentz et al., 2020; Docherty et al., 2020; Grasselli et al., 2020 ). Against this background, documenting and evaluating real-world experiences of care delivery is essential to inform future preparedness, particularly in resource-constrained settings (Dambadarjaa et al., 2022). The present study provides a comparative assessment of how COVID-19 pneumonia was managed at Mongolia’s two highest-ranked referral facilities, the Mongolia-Japan Hospital and the National Center for Communicable Diseases. In sharing these findings, we aim to contribute to global pandemic learning and to inform health-system strengthening in comparable low- and middle-income settings.

## Methods

### Study design and participants

Patient records from the Mongolia-Japan Hospital (MJH) and the National Center for Communicable Diseases (NCCD), the two largest tertiary referral hospitals in Mongolia, were used to conduct a retrospective cross-sectional study. The analysis covered the period from 18 April to 31 December 2021, which coincided with an unprecedented national surge in COVID-19 pneumonia. Patients were eligible for inclusion if they were (1) aged 18 years or older; (2) had a positive SARS-CoV-2 polymerase chain reaction (PCR) or antigen test; (3) had a diagnosis of COVID-19 pneumonia; and (4) had received pneumonia treatment in accordance with a local and/or international guideline (MOH, 2021; WHO, 2021). Patients with missing data on baseline clinical status or treatment regimens were excluded, as were those transferred to another facility before completing their course of care. Of the 2000 patients eligible during the study period, 1012 met the inclusion criteria-482 at MJH and 530 at NCCD.

### Data collection and variables

Anonymised data were extracted from hospital electronic health records and centralised health information registries. The variables collected included baseline demographics (age, sex, and body mass index); clinical status on admission (respiratory rate, pulse, oxygen saturation (SpO_2_), arterial blood pressure, temperature, and severity classified according to World Health Organization [WHO] criteria); and documented comorbidities (e.g. hypertension, diabetes mellitus, and other chronic conditions) (WHO, 2019). All pharmacological therapies administered during hospitalisation were recorded, with particular attention to broad-spectrum antibiotics, antivirals, corticosteroids, and anticoagulants (MOH, 2021; WHO, 2022b). For each agent, the generic name, dosage, and route of administration were documented. All data were checked for internal consistency, plausibility, and completeness before analysis.

### Drug classification and utilisation analysis

Antibiotics were grouped into pharmacological classes, including cephalosporins, macrolides, fluoroquinolones, and others (Langford et al., 2021; Calderon et al., 2023). The ‘other’ category comprised agents such as linezolid, rifaximin, nitrofurantoin, sulfasalazine, and metronidazole (WHO, 2023). The antivirals assessed were umifenovir, remdesivir, and favipiravir (Huang et al., 2021; WHO, 2022b). Monoclonal antibody therapy, specifically tocilizumab, was recorded separately as an immunomodulatory regimen (WHO, 2022b; Siemieniuk et al., 2020). The number, frequency, and specific multi-drug combinations of antimicrobials prescribed per patient were recorded to characterise institutional empirical prescribing under pandemic strain.

### Statistical analysis

Data were summarised using descriptive statistics and compared between the two hospitals. Categorical variables are presented as frequencies and percentages, and continuous variables as means with standard deviations (SD). Categorical variables were compared using the chi-square test (or Fisher’s exact test where appropriate) and continuous variables using the independent-samples t-test. A two-sided *p*-value below 0.05 was considered statistically significant. All analyses were performed in SPSS (version 25.0) and ethics approval was obtained from the Research Committee of Mongolian National University of Medical Sciences (protocol No2021/03/13).

## Results

### Demographic and clinical characteristics

A total of 1012 patients with COVID-19 pneumonia were included, 482 at MJH and 530 at NCCD. Their demographic and clinical characteristics, together with overall patterns of medication use, are summarised in Table 1.

**Table 1.** Demographic profile, clinical parameters, and comorbidity patterns among hospitalised COVID-19 pneumonia patients at MJH and NCCD (*n* = 1012).

Variables	Category	MJH (*n* = 482)	NCCD (*n* = 530)	*p* value
Age (mean, SD)		52.9 (16.8)	51.2 (16.7)	0.107
Age group (years) *n* (%)	<19	7 (1.4)	9 (1.6)	
	20–29	26 (5.3)	43 (8.1)	
	30–39	90 (18.6)	94 (17.7)	0.225
	40–49 50–59	66 (13.6) 127 (26.3)	91 (17.1) 117 (22.0)	
	>60	166 (34.4)	176 (33.2)	
Gender n (%)	Female	287 (59.5)	339 (64.0)	0.167
	Male	195 (40.5)	191 (36.0)	
Number of patients with comorbidities n (%)	Hypertension	182 (37.8)	67 (12.6)	<0.001
	Diabetes Other None	95 (19.7) 117 (24.2) 88 (18.2)	32 (6.0) 27 (5.0) 404 (76.2)	
COVID-19 severity n (%)	Mild	43 (8.9)	67 (12.6)	
	Moderate	342 (70.9)	376 (70.9)	<0.001
	Severe	80 (16.5)	51 (9.6)	
	Critical	17 (3.5)	36 (6.7)	
Clinical characteristics (mean, SD)	SpO_2_	94.2 (3.6)	95.0 (3.6)	<0.001
	Respiration rate	20.2 (3.3)	20.3 (3.1)	0.620
	Pulse	83.3 (12.6)	83.5 (13.5)	0.807
	Temperature	36.6 (0.7)	36.7 (0.8)	0.034
	Systolic pressure	133.9 (19.7)	122.2 (19.8)	<0.001
	BMI	28.0 (5.8)	27.0 (5.5)	0.005
Body mass index group *n* (%)	<18.5	3 (0.6)	19 (3.5)	
	18.5–24.9	157 (32.5)	176 (33.2)	0.001
	25–29.9	170 (35.3)	205 (38.6)	
	>30	152 (31.5)	130 (24.5)	
Number of patients positive tested	CRP	399 (82.7)	17 (3.2)	<0.001
	D dimer	46 (9.5)	10 (1.9)	<0.001
	PCR	482 (99.8)	529 (99.8)	1.000
Number of patients chest examination	X-ray	447 (92.7)	530 (100)	<0.001
	CT	237 (49.1)	529 (99.8)	<0.001
Hospital stays (mean day, SD)		13.5 (4.9)	13.0 (4.7)	0.099
Number of patients medicines prescribed (pharmacologic group)	Antibiotics	430 (89.2)	461 (86.9)	0.320
	Antivirals	361 (74.8)	490 (92.2)	<0.001
	Antifungals	49 (10.1)	33 (6.2)	0.029
	Glucocorticoids	305 (63.2)	279 (52.6)	<0.001
	Anticoagulant	326 (67.6)	253 (47.7)	<0.001
	Antiplatelet	375 (77.8)	280 (52.8)	<0.001
Total medicines used per patients (mean, SD)		10.6 (3.7)	9.1 (4.4)	<0.001

The mean age of the cohort was 52.0 years (SD 16.8), and 32% (324/1012) of patients were aged 61 years or older. Women accounted for 61.9% (626/1012) of the sample. The mean body mass index (BMI) was 27.5 kg/m² (SD 5.6), and 27.8% (282/1012) of patients were obese (BMI > 30 kg/m²).

Hypertension was the most common comorbidity, 24.6% (249/1012), followed by diabetes mellitus, 12.5% (127/1012). According to the WHO classification, most patients had moderate disease, 71.7% (726/1012), whereas 12.0% (121/1012) had severe, 11.2% (113/1012) mild, and 5.1% (52/1012) critical disease. Admission parameters – oxygen saturation (SpO₂), respiratory rate, heart rate, body temperature, and systolic blood pressure – did not differ significantly between the two hospitals (*p* < 0.05).

Diagnostic testing was near-complete: SARS-CoV-2 PCR was performed in 99.8% (1010/1012) of patients and antigen testing in the remaining 2, while 96.6% (978/1012) underwent chest radiography and 76.7% (776/1012) underwent chest computed tomography.

### Patterns of antibiotic prescription

Antibiotics were prescribed to 87.6% (887/1012) of participants. The distribution of antibiotic and antiviral prescribing across the two hospitals is summarised in Table 2, and the dual and triple antibiotic combinations are detailed in Table 3 (Alshaikh et al., 2022; Calderon et al., 2023; Granata et al., 2022; Langford et al., 2021; Rawson et al., 2021). Cephalosporins were the most commonly prescribed class, 89.3% (904/1012), followed by macrolides, 36.5% (369/1012), and fluoroquinolones, 33.5% (339/1012) (Calderon et al., 2023). Less frequently used agents included penicillins, 8.7% (88/1012), carbapenems, 4.0% (40/1012), aminoglycosides, 2.0% (20/1012), tetracyclines, 2.0% (20/1012), glycopeptides, 0.6% (6/1012), and oxazolidinones, 1.2% (12/1012) (Granata et al., 2022; Langford et al., 2021; Rawson et al., 2021).

**Table 2.** Antibiotic and antiviral prescription pattern by pharmacologic class, combination use, and route of administration among COVID-19 inpatients at MJH and NCCD.

Variable	Category	MJH (n = 482) *n* (%)	NCCD (*n* = 530) *n* (%)	*p* value
**Antibiotic class**				
	Penicillin (e.g. amoxicillin–clavulanate)	72 (14.9)	16 (3.3)	<0.001
	Cephalosporins (e.g. ceftriaxone, cefepime)	422 (87.6)	482 (100.0)	0.100
	Fluoroquinolones (e.g. levofloxacin, moxifloxacin)	189 (39.2)	150 (31.1)	<0.001
	Macrolides (azithromycin, clarithromycin)	285 (59.1)	84 (17.4)	<0.001
	Carbapenems (meropenem, imipenem)	24 (5.0)	16 (3.3)	0.151
	Aminoglycosides (gentamicin)	0 (0.0)	20 (4.1)	<0.001
	Tetracyclines (doxycycline)	20 (4.1)	0 (0.0)	<0.001
	Glycopeptides (vancomycin)	3 (0.6)	3 (0.6)	1.000
	Oxazolidinones (linezolid)	12 (2.5)	0 (0.0)	<0.001
**Antibiotics prescribed per patient**				
	None	52 (10.7)	69 (13.0)	<0.001
	One antibiotic	116 (24.0)	273 (51.5)	
	Two antibiotics	239 (49.5)	167 (31.5)	
	Three or more antibiotics	75 (15.5)	21 (3.9)	
**Route of administration (doses)**				
	Oral	444 (43.1)	198 (25.6)	<0.001
	Parenteral	587 (56.9)	576 (74.4)	
**Antiviral agent**				
	Umifenovir	172 (35.7)	356 (67.2)	<0.001
	Remdesivir	76 (15.8)	130 (24.5)	<0.001
	Favipiravir	138 (28.6)	38 (7.2)	<0.001
	Tocilizumab	2 (0.4)	0 (0.0)	0.227
**Antivirals prescribed per patient**				
	None	127 (26.3)	45 (8.5)	<0.001
	One antiviral	310 (64.3)	293 (55.3)	
	Two antivirals	41 (8.5)	175 (33.0)

**Table 3.** Frequency and distribution of dual and triple antibiotic combinations used in hospitalised COVID-19 patients at MJH and NCCD.

Antibiotic combination	MJH (*n* = 239) *n* (%)	NCCD (*n* = 167) *n* (%)	*p* value
**Two antibiotics**			
Beta-lactam + beta-lactam	10 (4.1)	10 (6.0)	0.553
Beta-lactam + aminoglycoside	–	10 (6.0)	<0.001
Beta-lactam + macrolide	132 (55.2)	48 (28.7)	<0.001
Beta-lactam + tetracycline	5 (2.0)	–	0.081
Beta-lactam + quinolone	63 (26.3)	93 (55.6)	<0.001
Beta-lactam + glycopeptide	–	2 (1.2)	0.169
Beta-lactam + other	4 (1.6)	2 (1.2)	1.000
Macrolide + quinolone	23 (9.6)	1 (0.5)	<0.001
Glycopeptide + other	2 (0.8)	–	0.515
Other + other	1 (0.4)	–	1.000
**Three antibiotics**			
Beta-lactam + beta-lactam + quinolone	2 (2.6)	5 (23.8)	0.005
Beta-lactam + beta-lactam + macrolide	3 (4.0)	1 (4.7)	1.000
Beta-lactam + aminoglycoside + quinolone	–	5 (23.8)	<0.001
Beta-lactam + aminoglycoside + macrolide	–	2 (9.5)	0/046
Beta-lactam + macrolide + quinolone	60 (80.0)	8 (38.0)	<0.001
Beta-lactam + macrolide + tetracycline	7 (9.3)	–	0.341
Beta-lactam + tetracycline + other	3 (4.0)	–	1.000

Combination therapy was common: 35.2% (356/1012) of patients received a single agent, 30.5% (309/1012) two agents, and 21.9% (222/1012) three or more (Alshaikh et al., 2022; Calderon et al., 2023; Granata et al., 2022; Lai et al., 2021). The most frequent dual regimen was a beta-lactam plus a macrolide, which accounted for 44.3% (180/406) of combination therapy and was used more often at MJH 55.2% (132/239) than at NCCD 28.7% (48/167). The next most frequent was a beta-lactam plus a quinolone, 38.4% (156/406), which was more common at NCCD 55.6% (93/167) than at MJH 26.3% (63/239). Among patients receiving three antibiotics, the combination of a beta-lactam, a macrolide, and a quinolone was the most frequent, 70.8% (68/96) (Dambadarjaa et al., 2022; Ibn-Mohammed et al., 2021; WHO, 2020b).

Microbiological cultures were obtained from only 21.5% (218/1012) of patients; of these, 34.4% (75/218) were positive, most commonly for gram-negative organisms, followed by *Staphylococcus spp*.

### Antiviral prescription patterns

Antivirals were administered to 90.1% (912/1012) of patients. Umifenovir was the most frequently prescribed agent, 52.2% (528/1012), followed by remdesivir, 20.4% (206/1012), and favipiravir, 17.4% (176/1012) (Infectious Diseases Society of America [IDSA], 2022; Siemieniuk et al., 2020; WHO, 2022b). Tocilizumab was used in only 2 patients, 0.4% (2/4892). Most patients received either one antiviral, 59.6% (603/1012), or two, 21.3% (216/1012) (IDSA, 2022; Pilkington et al., 2020; Siemieniuk et al., 2020; WHO, 2022b). Parenteral administration accounted for 64.4% (1163/1805) of antibiotic doses and was less frequent at MJH, 56.9% (587/1031), than at NCCD (74.4%, 576/774), indicating institutional variation in practice.

## Discussion

During the study period, most patients admitted with COVID-19 pneumonia at the two referral hospitals had moderate disease, and a high proportion were male. This pattern is consistent with hospital cohorts reported from other countries, including China (Jin et al., 2020; Peckham et al., 2020; Scully et al., 2020; Williamson et al., 2020). In line with global findings, comorbidities were common, particularly hypertension and diabetes mellitus, both of which are established risk factors for adverse outcomes (Barron et al., 2020; Grasselli et al., 2020; Zhou et al., 2020). Hypertension was recorded more often at MJH than at NCCD, most likely reflecting differences in the two hospitals’ populations as shown on national registry (Dambadarjaa et al., 2022; MOH, 2023).

Antibiotics were prescribed to nearly nine of every ten patients, 87.6% (887/1012), all of whom had laboratory-confirmed SARS-CoV-2 infection and pneumonia diagnosed largely on clinical grounds. High antibiotic use was documented previously in Mongolia: a national survey conducted in early 2019 reported prescription rates of 27–95% in secondary and tertiary hospitals (MOH, 2021). In addition to this high baseline prescribing, local guidelines adapted from international COVID-19 recommendations may have further encouraged antibiotic use, with or without bacteriological confirmation, in patients with pneumonia (MOH, 2021; WHO, 2021). In our study, a high proportion of patients with elevated inflammatory markers, such as C-reactive protein and procalcitonin, may have been an additional driver, alongside clinical presentation. This pattern mirrors early international experience, when broad-spectrum antibiotics were widely used to treat or empirically cover suspected bacterial co-infection despite limited microbiological testing evidence (Alshaikh et al., 2022; Calderon et al., 2023; Granata et al., 2022; Langford et al., 2021; Rawson et al., 2021). Combination antibiotic therapy was frequent, with dual and triple regimens used from several different classes. A beta-lactam plus a macrolide predominated at MJH, whereas a beta-lactam plus a quinolone was more common at NCCD; both combinations were recommended by WHO and other guidelines during the study period (COVID-19 Clinical Management: living guidance, 25 January 2021 – PAHO/WHO | Pan American Health Organization). NCCD also used triple therapy combining a beta-lactam, a macrolide, and a quinolone on occasion. Given the limited access to bacteriological testing and the resource constraints of the pandemic, such empirical multi-drug therapy was often difficult to avoid, as reported in other settings (Alshaikh et al., 2022; Hossain et al., 2023). Nonetheless, the high rate of antibiotic combinations underscores the need for routine microbiological testing and post-prescription review to promote rational, evidence-based use (Hossain et al., 2023). Strengthening real-time microbiology laboratory capacity is a necessary precondition for effective antibiotic stewardship in resource-limited hospitals such as our setting. Although clinical outcomes were not included in our study, patient s’ comorbidities, body mass index, etc., may have influenced patient outcome and future studies linking drug prescription and outcomes are therefore warranted (Petrilli et al., 2020).

Antiviral use mirrored the pattern seen for antibiotics: almost every patient (90.1%, 912/1012) received at least one antiviral, consistent with the national and WHO recommendations at the time during COVID-19 (MOH, 2021; WHO, 2022a). Umifenovir and favipiravir were prescribed most often, largely reflecting their local availability, oral administration, and low cost during the study period. Remdesivir, the only antiviral with a conditional recommendation for selected hospitalised patients in the WHO guideline, was used far less often, most likely because of its higher cost, parenteral administration, and limited supply (Adab, 2022). Tocilizumab, recommended for severe or critical disease, was given to only 2 patients, suggesting limited access to newer, evidence-based therapies during the surge. Taken together, these findings indicate that antiviral selection was driven mainly by availability and affordability. From a policy standpoint, national essential-medicines lists and procurement should be updated quickly as international guideline updates (WHO, 2022a, 2022b).

Several limitations should be considered when interpreting our findings. First, this was a retrospective study that used descriptive statistics to characterise prescribing patterns; we did not perform inferential or multivariable analyses and therefore cannot identify the factors that independently drove inter- and intra-hospital variation in prescribing. Second, as with any retrospective study, our analysis was limited by the availability, accuracy, and completeness of routinely recorded data that may have limited the representativeness of the findings. Third, we did not link prescribing to clinical outcomes such as length of stay or mortality, we cannot comment on the appropriateness or effectiveness of the overprescription. Despite these limitations, the study provides useful real-world information on inpatient prescribing for COVID-19 pneumonia, highlights opportunities to improve the management of medical records and pharmaceutical resources and offers lessons that may be transferable to comparable resource-limited settings during future public health emergencies.

## Conclusion

In summary, antibiotic and antiviral prescribing among patients hospitalised with COVID-19 pneumonia at two Mongolian tertiary hospitals was extensive and varied markedly between institutions. These patterns, overprescription of broad-spectrum antibiotics and combinations along with extensive antiviral treatment, highlight the need to strengthen antimicrobial stewardship at each hospital . At the policy level, we recommend improving hospital-based antimicrobial stewardship programmes supported by timely microbiology testing. In addition, further classifying and monitoring antibiotic use within the WHO AWaRe framework, and aligning national treatment guidelines and medicines lists with international evidence are essential. Implementing these measures would promote the better use of medicines, help contain antimicrobial resistance, and strengthen preparedness for future public health emergencies in Mongolia and comparable low- and middle-income settings.

## Data Availability

The dataset supporting the conclusions of this article is included within the article and its additional files.
